# Influence of Density Gradient on the Compression of Functionally Graded BCC Lattice Structure

**DOI:** 10.3390/ma16020520

**Published:** 2023-01-05

**Authors:** Yuxiang Lin, Wentian Shi, Xiaohong Sun, Shuai Liu, Jihang Li, Yusheng Zhou, Yifan Han

**Affiliations:** School of Artificial Intelligence, Beijing Technology and Business University, Beijing 100048, China

**Keywords:** functional gradient lattice structures, BCC, compression properties, Ti-6Al-4V, selective laser melting, finite element analysis

## Abstract

In this paper, five grading functional gradient lattice structures with a different density perpendicular to the loading direction were proposed, and the surface morphology, deformation behavior, and compression properties of the functional gradient lattice structures prepared by selective laser melting (SLM) with Ti-6Al-4V as the building material were investigated. The results show that the characteristics of the laser energy distribution of the SLM molding process make the spherical metal powder adhere to the surface of the lattice structure struts, resulting in the actual relative density of the lattice structure being higher than the designed theoretical relative density, but the maximum error does not exceed 3.33%. With the same relative density, all lattice structures with density gradients perpendicular to the loading direction have better mechanical properties than the uniform lattice structure, in particular, the elastic modulus of LF, the yield strength of LINEAR, and the first maximum compression strength of INDEX are 28.99%, 16.77%, and 14.46% higher than that of the UNIFORM. In addition, the energy absorption per unit volume of the INDEX and LINEAR is 38.38% and 48.29% higher, respectively, than that of the UNIFORM. Fracture morphology analysis shows that the fracture morphology of these lattice structures shows dimples and smooth planes, indicating that the lattice structure exhibits a mixed brittle and ductile failure mechanism under compressive loading. Finite element analysis results show that when the loading direction is perpendicular to the density gradient-forming direction, the higher density part of the lattice structure is the main bearing part, and the greater the density difference between the two ends of the lattice structure, the greater the elastic modulus.

## 1. Introduction

Based on the optimized pore-size distribution and relative density, functional gradient lattice structures (FGLs) are of increasing interest for their potential to enable the tailoring of structural responses and development of multifunctional applications and are widely used in the biomedical and aerospace spheres [1,2,3]. The lattice structure can be divided into a stochastic lattice structure and non-stochastic lattice structure [4], such as functionally graded metal syntactical foams [5,6,7], closed-cell foams [8,9,10], and open-cell foams [11], which have random distribution characteristics and are defined as a stochastic lattice structure; meanwhile, the lattice structure formed by repeating a unit cell arrangement is classified as a non-stochastic lattice structure. However, it is difficult for traditional manufacturing techniques, such as melt gas injection [12], physical vapor deposition [13], and sheet metal technology [14], to produce complex lattice structures with high precision and costly manufacturing. Selective laser melting (SLM) technology, a type of additive manufacturing technology, is a powder bed-based melting technique commonly used to fabricate lattice structures with high precision due to its fine feature resolution and extensive mechanical support during the processing of the surrounding compacted-powder bed [15].

Currently, three approaches are commonly used to achieve non-stochastic lattice structures with gradients: (1) varying the strut size of the unit cell [16,17,18,19]; (2) varying the size of the unit cell [19,20,21]; (3) combining different types of unit cells [22,23]. However, some grading methods may lead to weak layers or discontinuous unit cells, such as changing the unit cell size or combining different unit cells. However, these defects can be effectively avoided by changing the strut diameter to achieve the gradient, and therefore this method of realizing the gradient of lattice structure has been more widely adopted in the more recent research [24,25,26].

Investigations of non-stochastic FGLs by early researchers were usually limited to designing a gradient for the lattice structure and comparing it with a gradient-free (uniform) lattice structure [27,28,29]. Recently, however, researchers have noticed that the span of the gradient has different effects on the mechanical properties of non-stochastic FGLs. Han et al. [30] used pure titanium powder to fabricate five lattice structures with different density gradient spans with the same volume fraction in the distal layers by SLM, and the results showed that the smaller the gradient span, the better the mechanical properties of the lattice structure. Plocher et al. [19] used short carbon fiber-reinforced nylon materials to fabricate four body-centered cubic (BCC) lattice structures with different density gradient spans in a fused deposition modeling approach and found that the lattice structures with a larger density gradient span had significantly lower stiffness than the uniform lattice structure. It is worth noting that the density gradient mentioned is realized parallel to the loading direction. However, the mechanical properties of lattice structures with density gradients in the direction perpendicular to the loading direction have also been investigated by several researchers. Yang et al. [16] fabricated a Gyroid cell structure with a density gradient in the direction perpendicular to the loading direction using SLM with 316 L material. The results show that the density gradient perpendicular to the loading direction has a similar deformation behavior to that of the uniform density structure, but Young’s modulus, platform stress, and energy absorption capacity are better than that of the uniform density structure. Niknam et al. [31] fabricated three lattice structures with different density gradients perpendicular to the loading direction by stereolithography using an elastic resin, and the results showed that the stiffness and energy absorption capacity of the lattice structures with density gradients perpendicular to the loading direction are better than that of the uniform lattice structure, and the mechanical properties of these three lattice structures with different density gradients are also different. Nevertheless, the density gradient perpendicular to the loading direction has not been sufficiently investigated, especially for specimens fabricated on SLM using Ti-6Al-4V (TC4) alloy powder, and hence this is still a direction that needs to continue to be explored.

Therefore, based on TC4 alloy powder and the SLM fabrication technique, this study aims to try to investigate experimentally how different density gradient formation methods in the direction perpendicular to the loading direction affect the mechanical properties of lattices with the same relative density tested in compression. In this study, five different density gradient formation strategies were proposed based on BCC lattice structures by varying the strut diameter of the unit cell, and their morphology, dimensional accuracy, yield strength, elastic modulus, plateau stress, first maximum compressive strength, energy absorption, deformation behavior, and fracture surface were investigated. In conclusion, this work provides a valuable reference for the design of non-stochastic FGLs and provides insight into the design of non-stochastic FGLs in specific cases.

## 2. Material and Methods

### 2.1. Model Design and Manufacturing

**Figure 1:**
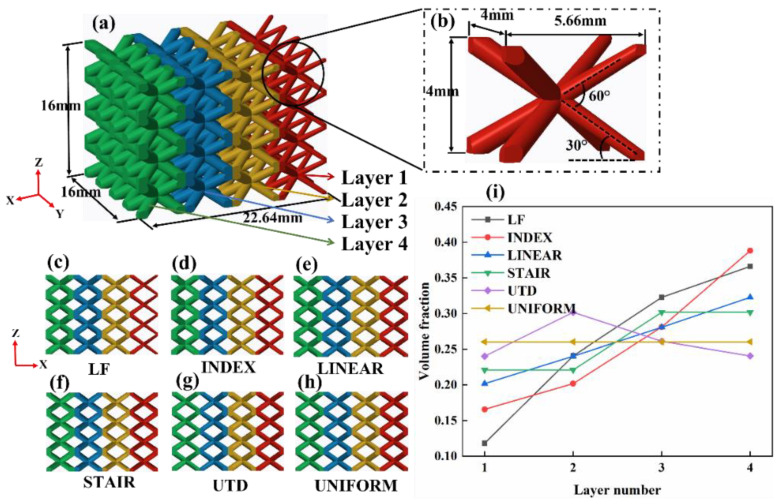
CAD-based configuration and geometry of the lattice structure (**a**) and unit cell (**b**), the front view of each lattice structure for different density gradients (**c**–**h**), and the volume fraction profiles corresponding to different layers of the lattice structure with different density gradients (**i**).

BCC FGLs were created using the Computer-Aided Design (CAD) software Creo Parametric^TM^ 3.0 (Boston, MA, USA). Each printed structure maintains a rectangular cross-section of 16 mm by 22.64 mm and a height of 16 mm. Depending on the configuration and design, 4 unit cells span the cross section and height. It should be noted that the strut diameter of the BCC lattice structure was designed to vary continuously along the strut, which is intended to avoid the appearance of weak layers or discontinuous unit cells, and based on this, five of the lattice structures with different density gradient spans were designed and named sequentially as LF, INDEX, LINEAR, STAIR, and UTD. This naming rule is based on the curves of different density gradient changes, such as LF indicating that the density gradient change is a logarithmic function type, INDEX representing the density gradient change as index type, LINEAR standing for the density gradient change is a linear type, STAIR and UTD representing the density gradient change as a stair type and increasing followed by decreasing type, respectively. For comparison purposes, uniform gradient lattice structures were also created and named UNIFORM. Figure 1 not only shows the volume fraction profiles corresponding to different layers of the lattice structure with different density gradients, and also shows the configuration and geometry of the lattice structure and unit cell as well as the front view of each lattice structure with different density gradients.

**Figure 2:**
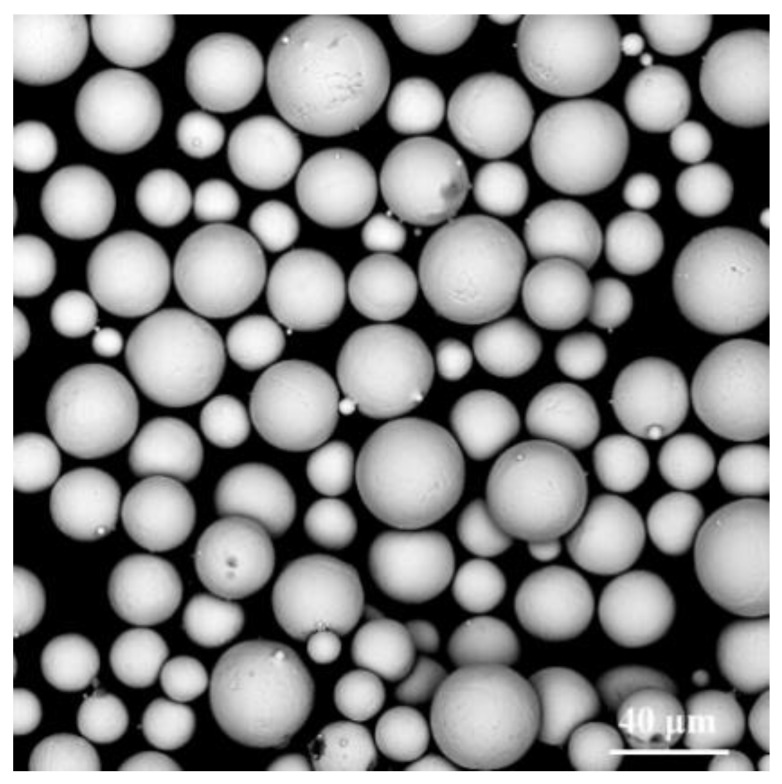
SEM images of TC4 metal powder morphology.

The specimens were printed with a Selective laser melting machine (AM400; Renishaw plc, London, UK), using TC4 titanium alloy powder (prepared by gas atomization method; FeiErkang Rapid Manufacturing Technology Co., Ltd, Wuxi, China). The particle size of TC4 titanium alloy powder is 15–53 µm (tested by Laser particle size analyzer, Cilas 990 DL; CILAS, Orleans, France), and its morphology and chemical composition are shown in Figure 2 and Table 1, respectively. According to the preliminary study of the SLM forming process parameters [32,33,34], the forming parameters were 230 W laser power, 100 μs exposure time, 50 μm point distance, 100 μm hatch spacing, and 50 μm layer thickness. After the specimens were formed, the fabricated lattice structures were separated from the platform plate of the SLM machine by an EDM (electrical discharge machining) wire-cutting process on a CMNE machine (Beijing, China), and no heat treatment was applied to the sample. It is important to note that, for each type of lattice structure, 5 samples were manufactured for the subsequent tests.

### 2.2. Density Measurement and Morphology Characterization

The porosity of the lattice structure and the morphology of the struts after fabrication have a significant influence on the mechanical properties [35,36], and therefore these parameters were considered in this study. Archimedes’ drainage method was used to measure the porosity of all specimens [37]. The formula for calculating the lattice structure density ($\rho_{lattice}$) and porosity (φ) are as follows:

(1) $$\rho_{lattice}=\frac{W_{air}}{W_{air}-W_{water}}\cdot \rho_{water}$$

(2) $$\varphi =1-\frac{\rho_{lattice}}{\rho_{S}}$$

where $W_{air}$ and $W_{water}$ represent the specimen weights in air and water, respectively. $\rho_{water}$ is set at 1 g/cm^3^ as the pure water density at an ambient temperature of 25 °C. $\rho_{s}$ is the theoretical density of the parent material TC4 (4.43 g/cm^3^). For each sample, the measurement was repeated three times. In addition, the optical microscope (OM, KEYENCE, VHX-600E, Osaka, Japan) was used to observe the morphology of the lattice structures after fabrication and quasi-static experimental tests. The surface morphology of the SLM fabricated the lattice structures before the deformation process and fracture surfaces’ morphology after the deformation underwent micro-morphological characterization using a Phenom XL Scanning Electron Microscope (SEM; Phenom XL, Amsterdam, The Netherlands). Additionally, to ensure the accuracy of the measurements and characterization, the lattice structure was ultrasonically cleaned with anhydrous ethanol for 20 min before using the SEM and measuring the porosity of the lattice structures.

### 2.3. Quasi-Static Experimental Testing

Uniaxial compression tests were performed with an electronic universal testing instrument (UTM5305; Shenzhen, China) equipped with a 100 KN load capacity. According to the ISO13314:2011 standard [38], the loading rate of the crosshead was set at a strain rate of 0.001/s^−1^. The compression experiments were performed at room temperature (25 °C) and all specimens were centrally placed between two steel plates. The compressive stress was determined by dividing the load value obtained by the crosshead by the effective area of the lattice structure, and the compressive strain was determined by dividing the displacement of the crosshead by the actual height of the lattice structure, from which a nominal stress–strain curve was plotted from the average value. Unless specifically stated, the mechanical property values for all samples were calculated from nominal stress–strain curves. To avoid the effects of building orientation, all compression tests were conducted along the building direction [39]. The experiment was terminated when the displacement of the crosshead was about 10 mm. In order to observe the deformation process of different lattice structures during quasi-static compression, the front side of the lattice structure (parallel to the global X-Z plane as shown in Figure 1) was recorded using a mobile camera.

### 2.4. Statistical Analysis

Each sample was subjected to five repeated experiments, and the data of each experiment were normalized relative to the control experiment and expressed as the average of five repeated standard deviations. Statistical analysis was 95% confidence interval, and one-way ANOVA was adopted.

## 3. Results and Discussion

### 3.1. Surface Morphologies of the As-Built Lattice Structure

**Figure 3:**
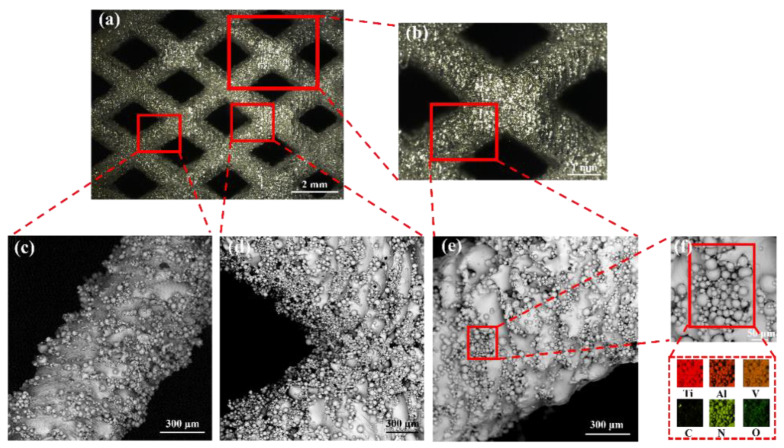
Optical microscope (OM) observation of the macroscopic morphology of the lattice structure (**a**,**b**); SEM micrographs of the struts with different diameters (**c**,**e**) and at the nodes (**d**), and energy spectrum analysis of the spherical particles on the surface of the strut (**f**).

Figure 3 shows the macroscopic morphology of the representative lattice structure observed by OM (a,b) and the microscopic morphology of struts with different diameters observed by SEM (c–e), and the energy spectrum analysis of spherical particles on the surface of the strut (f). It can be seen that a large number of unmelted or partially melted spherical particles were presented on the struts and at the joints of the struts, which was also found in previous studies [40,41]. The spherical particles adhering to the lattice structure’s struts will increase the overall mass of the lattice structure. Moreover, when measuring the mass of the lattice structure in water, it is smaller than the actual one due to the presence of bubbles inside the lattice structure. This would explain the higher relative density of each fabricated lattice structure compared to the designed value shown in Table 2. However, the maximum error does not exceed 3.33%, and no defects such as deformation and cracking were found for different strut sizes and at the joints of the struts, which demonstrates that these unsupported lattice structures can be successfully fabricated using SLM. The particle size analysis showed that the particle size distribution of the spherical particles attached to the struts was similar to that of the TC4 titanium alloy powder particles of the molding material, with most of the particles attached to the struts having a particle size distribution between 15 and 53 μm. In addition, the energy spectrum analysis of these spherical particles also showed that the spherical particles attached to the struts were mainly caused by the adhesion of partially molten TC4 titanium alloy metal powder particles on the surface of the struts. The reason for this phenomenon is due to the characteristics of the SLM manufacturing process, as the laser energy in the center of the laser spot is the highest, and the surrounding energy gradually decreases, so that the powder around the melt pool is not sufficiently melted, and thus the powder will adhere to the surface of the cured material after cooling. Both the larger and smaller strut diameter struts have a large number of spherical particles attached, but in general, the diameter of the relatively larger struts has fewer spherical particles attached to them than the smaller ones. It can, perhaps, be illustrated that the accuracy of the strut-based lattice structures with larger strut diameters manufactured by SLM is higher than that of the smaller strut diameters. The same phenomenon was found by Al-Saedi et al. [18], who fabricated the lattice structure of Al-12Si aluminum alloy with gradients using SLM and found that the error between the actual and design dimensions of the relatively large diameter strut was smaller than that of the smaller diameter strut. As can be seen from the SEM images in Figure 3, the stair-stepping effect is clearly evident, and these observations are also highlighted in [42]. The presence of the stair-stepping effect is likewise responsible for the roughness of the strut surface and the severe sticky powder phenomenon.

### 3.2. Quasi-Static Mechanical Test

#### 3.2.1. Mechanical Properties

**Figure 4:**
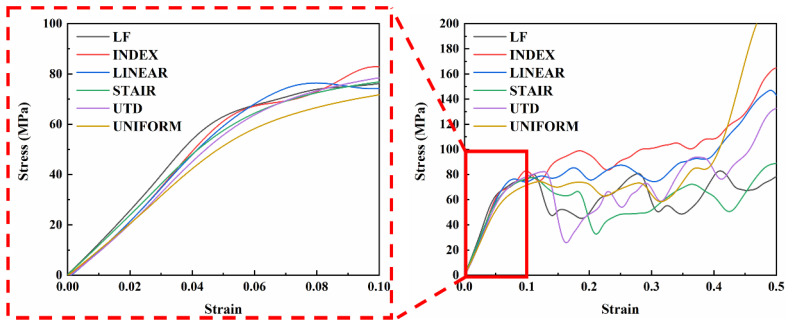
Experimentally determined compressive stress–strain curves for the fabricated lattice structures with different gradients and uniform lattice structures.

Figure 4 shows the compressive stress–strain curves of the lattice structure manufactured by SLM with different density gradients and uniform gradients perpendicular to the loading direction after the quasi-static compression test. For each different lattice structure, the first maximum compressive strength (defined as the first local maximum of the nominal stress–strain curve), the elastic modulus (defined as the initial linear slope in the nominal stress–strain curve), the compressive yield strength (obtained by the 0.2% offset method), and the plateau stress (defined as the average stress value in the strain range of 20% to 40%.), in accordance with ISO13314:2011 standard [38], are given in Table 3.

As can be seen in Figure 4, all types of lattice structures have similar characteristics to the stress–strain curves of ordinary metallic cellular solids, i.e., the linear elastic region, the long plateau region, and the densification region [43]. At low strains, both the LF, INDEX, and LINEAR samples show areas of multiple failures and show slight troughs in specific regions of the graph (the strain value between 0.06 and 0.1). This behavior is related to the brittle fracture of a small number of struts in the lattice structure, the failure behavior of the lattice structure will be described in detail in Section 3.2.3. However, as the strain increases, the compressive stress–strain curve of LF shows several sharp decreases in stress, while the compressive stress–strain curves of INDEX and LINEAR are flatter. The sharp drop of stress indicates the sudden failure of the structure, but for INDEX and LINEAR structures, it may be due to different gradient forming methods that these two structures have relatively stable failure forms to some extent. It can be seen in Figure 4 that when the strain is about 0.2, except for UTD (the stress of UTD has dropped sharply when the strain is lower), the stress of other lattice structures has dropped to some extent. The typical brittle diagonal shear fracture of the lattice structure in the early stage is the main reason for the sharp drop in stress. This may indicate that the deformation mode of these specimens was stretching-dominated. The occurrence of this sharp drop in stress is related to the design of the lattice structures and the TC4 alloy material used to fabricate the lattice structures. Ge et al. [44] fabricated two different lattice structures from TC4 titanium alloy and found that one lattice structure showed a sharp drop in stress in the plateau region during compression, while the other lattice structure showed a gentle change in stress. De Pasquale et al. [45] fabricated two different lattice structures using 316 L material, and both lattice structures showed no significant drop in stress in the plateau region under compressive loading. This is because the TC4 alloy is a brittle material, while 316 L alloy is a plastic material. The fracture of the struts and shear bands resulted in a sharp drop in stress, while the layer-by-layer failure of the lattice structure caused this phenomenon to occur repeatedly.

As seen in Table 3, the lattice structure with a density gradient perpendicular to the loading direction has a better elastic modulus, yield strength, and first maximum compressive strength than the uniform lattice structure. In particular, the elastic modulus of LF, the yield strength of LINEAR, and the first maximum compression strength of INDEX is 28.99%, 16.77%, and 14.46% higher, respectively, than that of the UNIFORM. The lattice structure with gradients perpendicular to the loading direction can significantly increase its strength compared to the uniform lattice structure with similar porosity. However, the plateau stress of UNIFORM is better than that of LF, STAIR, and UTD. The plateau stress will mainly affect the energy absorption of the lattice structure, which is discussed in detail in Section 3.2.2.

#### 3.2.2. Energy Absorption

**Figure 5:**
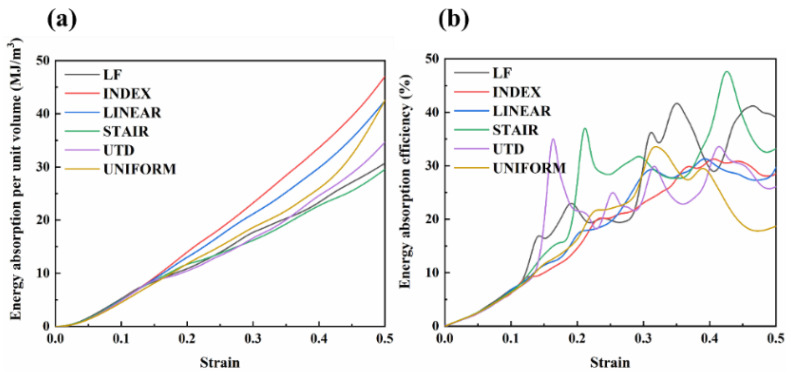
Energy absorption properties of different lattice structures; (**a**) energy absorption per unit volume; (**b**) energy absorption efficiency.

Analyzing the energy absorption capacity of different lattice structures can provide insights into the application of such structures to specific applications. In this study, the energy absorption per unit volume is defined as the strain value at the end of the plateau stress and the area enclosed by the nominal stress–strain curve; it is therefore defined by Equation (3), while the energy absorption efficiency is calculated by Equation (4). It is important to note that there are various methods for determining the starting strain for densification, such as the energy efficiency method [46,47] and 1.3 times the plateau stress [38]. However, in this work, the densification starting strain was determined using 1.3 times the platform stress according to ISO13314:2011 standard [38]:

(3) $$W=\int_{0}^{\varepsilon }\sigma d\varepsilon$$

(4) $$\eta =\frac{\int_{0}^{\varepsilon }\sigma d\varepsilon }{\sigma \left(\varepsilon \right)}$$

where W and η represent the energy absorption per unit volume (MJ/m^3^) and energy absorption efficiency of the lattice structure, respectively. σ(ε) is the compressive stress at strain ε. Table 4 provides the energy absorption properties and densification onset strain values for each of the different lattice structures. Although the energy absorption per unit volume of LF and STAIR is not as good as that of UNIFORM, the energy absorption per unit volume of INDEX and LINEAR is 38.38% and 48.29% higher, respectively, than that of UNIFORM. It is also evident from Figure 5a that the energy absorption per unit volume of INDEX and LINEAR is significantly better than that of UNIFORM before reaching the onset strain of densification. The energy absorption curves for each different lattice structure present a different trend at strain values of approximately 0.15.

**Figure 6:**
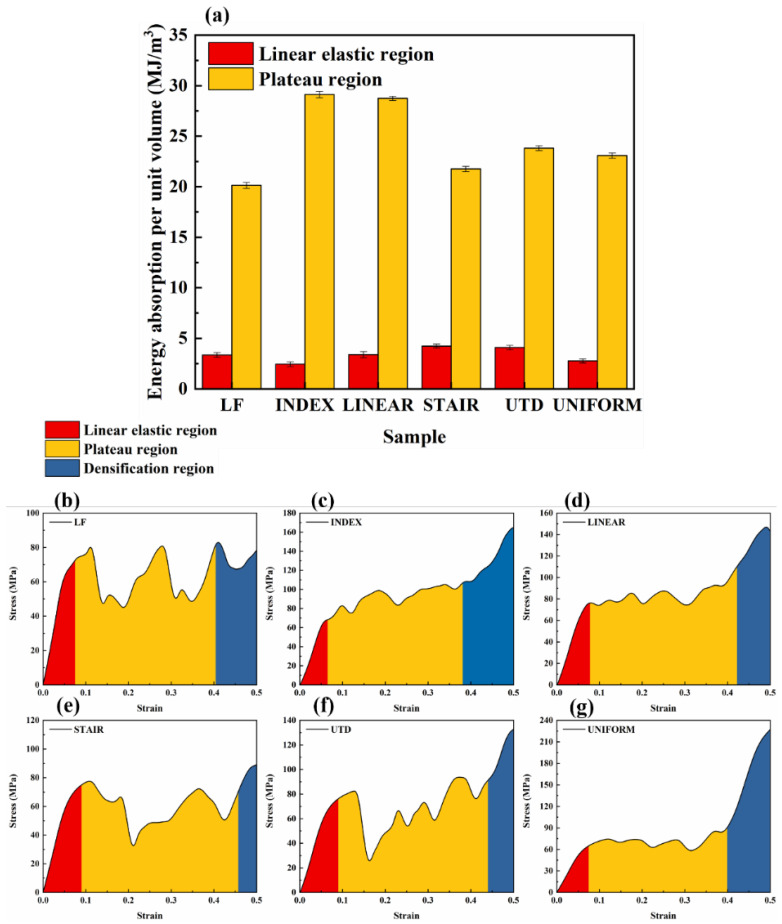
Energy absorption of different lattice structures in different regions (**a**) and schematic diagrams of the three deformation regions for different lattice structures (**b**–**g**).

Although the energy absorption curve of UTD is lower than that of UNIFORM at strains greater than 0.15, the final total energy absorption before reaching the onset strain of densification is slightly higher than that of UNIFORM. As can be seen in Figure 6a, the energy absorption per unit volume in the linear elastic region of UTD is 47.1% higher than that of UNIFORM, which can be attributed to the fact that at low strains, UTD has a higher first maximum compressive strength, yield strength, and elastic modulus than UNIFORM, resulting in UTD absorbing more energy at low strains than UNIFORM.

Similarly, it can be seen from Figure 6 that in general, lattice structures with a relatively flat plastic plateau region have a better energy absorption capacity, which is closely related to the design of the lattice structure. The energy absorption efficiency curves for the different lattice structures show a fluctuating increase until the onset densification strain is reached and a sharp decrease after reaching the onset densification strain. It is noteworthy that all lattice structures show a sharp downward trend after reaching the onset densification strain value, followed by a certain upward trend. This phenomenon occurs because the TC4 alloy is a brittle material and during the compression process after reaching the densification region, the mutual extrusion between the struts leads to further failure, resulting in fluctuations in the stress–strain curve for each lattice structure even after densification.

#### 3.2.3. Deformation Behavior under Quasi-Static Mechanical Test

**Figure 7:**
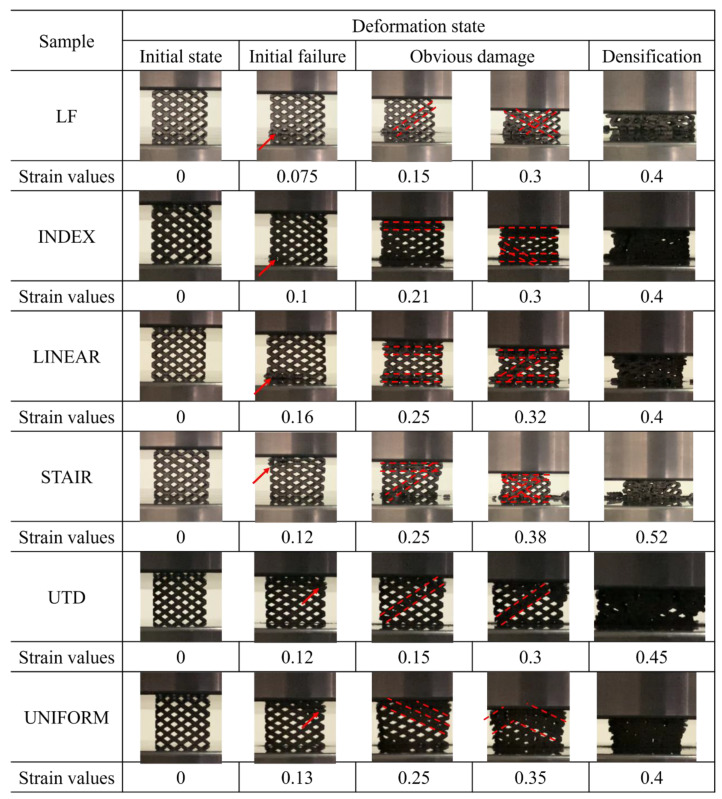
Deformation behavior of different lattice structures (The arrow indicates the location where the lattice structure first broke, and the dotted line indicates the diagonal shear break or crush of the lattice structure.).

The compressive deformation behavior of different lattice structures in quasi-static compression tests is presented in Figure 7.

The lower-left corner struts of the LF, INDEX, and LINEAR lattice structures are crushed at strains of approximately 0.075, 0.08, and 0.086, respectively, which are all indicated by arrows in Figure 7. The occurrence of this phenomenon is related to the design of the lattice structures, since the LF, INDEX, and LINEAR structures vary with different density gradients perpendicular to the loading direction (the density of the lattice structure decreases gradually from left to right in the direction shown in Figure 7). Since the lattice structure has a higher density on the left side and a relatively lower density on the right side, the lattice structure is subjected to a larger force on the left side when it is compressed, which leads to the premature fracture of the lower-left struts of the lattice structure. This is also confirmed by the finite element analysis in Section 3.4. However, as the strain increases, the deformation behavior of the LF lattice structure becomes different from that of the INDEX and LINEAR lattice structures. The LF lattice structure experienced two diagonal shear bands successively after the fracture of the lower-left corner struts, resulting in a sudden drop in compressive stress (indicated by dotted lines in Figure 7). For the INDEX and LINEAR lattice structures, the top and bottom layers of the lattice structure experienced compressional collapse before experiencing abrupt diagonal shear failure. This mode of failure will allow the struts in the central region of the lattice structure to be less affected by the loads applied to the lattice structure. This explains why, compared to the LF lattice structure, the INDEX and LINEAR lattice structures have a relatively flat and longer plateau region, as shown in Figure 4. The deformation behavior of the UTD and UNIFORM lattice structures at a strain of about 12% is relatively similar, unlike LF, INDEX, and LINEAR, where the fracture occurs in the upper-right corner of the lattice struts, while early strut failure in the STAIR lattice structure occurs in the upper-left corner. Combined with the model design in Figure 1, it can be seen that the lattice structure with density gradient tends to be damaged first in the part with a higher density under the action of the compressive load. With increasing strain, the deformation of LINEAR and STAIR seems to exhibit a Z-shape (shown by dotted lines in Figure 7), i.e., both the top and bottom layers of the lattice structure are crushed, and diagonal shear failure also occurs. In contrast, the UNIFORM lattice structure has a diagonal shear failure layer by layer at the upper-right corner. However, in general, the typical diagonal shear failure occurs for all lattice structures, but for the INDEX, LINEAR, and STAIR lattice structures, the lattice structure can sustain greater strains and absorb more energy due to the destruction of the top and bottom layers of the lattice structure before diagonal shear failure occurs. The sharp stress changes throughout the compressive loading process mean that the struts of the SLM-fabricated TC4 lattice structure are highly brittle. The fracture morphology of the struts enables further analysis of the failure mechanism of the lattice structure, which will be described in detail in Section 3.3.

### 3.3. Fracture Surface Characteristics

**Figure 8:**
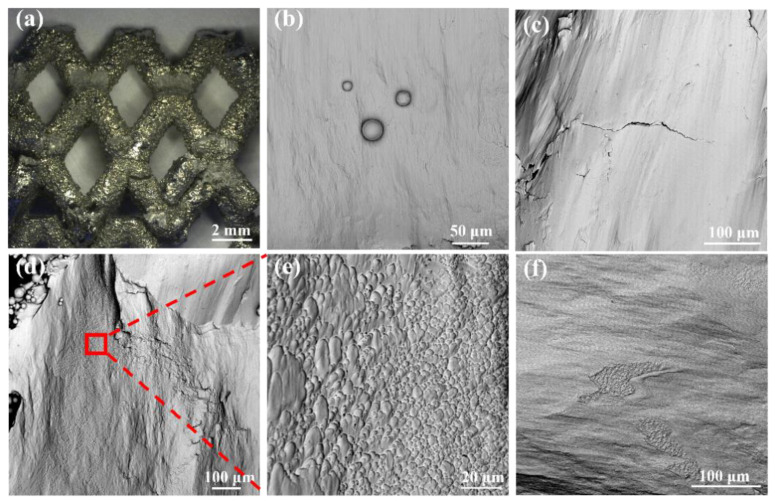
(**a**,**b**) are the macroscopic morphology of the representative specimen observed using OM and the splash defects observed by SEM, respectively, after the compression experiments. (**c**,**d**,**f**) show the nodal fracture morphology of UTD, INDEX, and UNIFORM lattice structures observed by SEM, respectively, where (**e**) is a local enlargement of the fracture morphology of INDEX lattice structure.

OM and SEM were used to observe the macroscopic and microscopic morphology of the fracture surface of the struts in order to study the mechanism of lattice structure damage, respectively. Since it is observed that the macroscopic morphology of the lattice structure with different ways of molding gradients perpendicular to the loading direction and the uniform lattice structure are almost the same after the quasi-static compression experiment. Therefore, the typical and representative macroscopic morphology is shown in Figure 8a. Most of the failures of the lattice structure occur at the nodes, which are related to the stress concentration. In addition, a fractured strut is found to be squeezed in the rhombus formed between the struts because of the mutual squeezing effect, which leads to the fracture of the strut near the node. Since the INDEX lattice structure and UTD lattice structure have relatively flat and relatively steep compressive stress–strain curves, respectively; therefore, SEM was used to observe the fracture morphology of INDEX and UTD lattice structure struts separately. In addition, for comparison, the fracture morphology of the UNIFORM lattice structure was also observed. Figure 8c–f show the fracture morphology of UTD, INDEX, and UNIFORM lattice structures at the nodes, respectively.

All three lattice structures show smooth brittle fractures with mostly stripy cleavage planes. It is also noteworthy that both INDEX and UNIFORM lattice structures show dimple and smooth plane morphological features. However, the dimple feature of the INDEX lattice structure is significantly more than that of UNIFORM. The presence of the dimple feature indicates that the lattice structure struts exhibit ductile fracture characteristics. This ductile fracture process ensures that the energy is continuously absorbed and thus can enhance the energy absorption capacity of the lattice structure [1], which is confirmed by the data in Table 4. It is worth noting that the energy absorption of the UNIFORM lattice structure is lower than that of the UTD. This is partly because the fracture morphology of the UNIFORM lattice structure shows dimple features, but the number is very small, resulting in minimal sustained energy absorption provided by the ductile fracture process; on the other hand, it is also because the UTD lattice structure has a different structural design from that of the UNIFORM, meaning that the UTD has a higher strength compared to the UNIFORM lattice structure, which also makes the energy absorption in the linear elastic region of the UTD lattice structure significantly higher than that of UNIFORM. The fracture morphology of the UTD (Figure 8c) also shows the presence of secondary cracks in the fracture. In the compression experiments, the lattice structure tends to expand outward during the loading process, resulting in high tension in the weak area in the middle of the strut, which leads to the phenomenon of secondary cracking in the fracture morphology. In general, the fracture morphology of the specimens at the nodes is mostly characterized by both smooth planes and dimples, which would indicate a mixed brittle and ductile failure mechanism. Some limitations should be acknowledged in this research. The presence of some unmelted metal powder on the strut surface and some splash defects at the fractures of the strut nodes can be observed in Figure 3c–e and Figure 8b, respectively. These defects will affect the mechanical properties of the lattice structure to some extent. By optimizing the SLM forming process parameters, manufacturing defects can be avoided to the maximum extent. The sticky powder phenomenon on the struts can be improved by post treatment, which should be fully investigated in the future.

### 3.4. Finite Element Analysis

**Figure 9:**
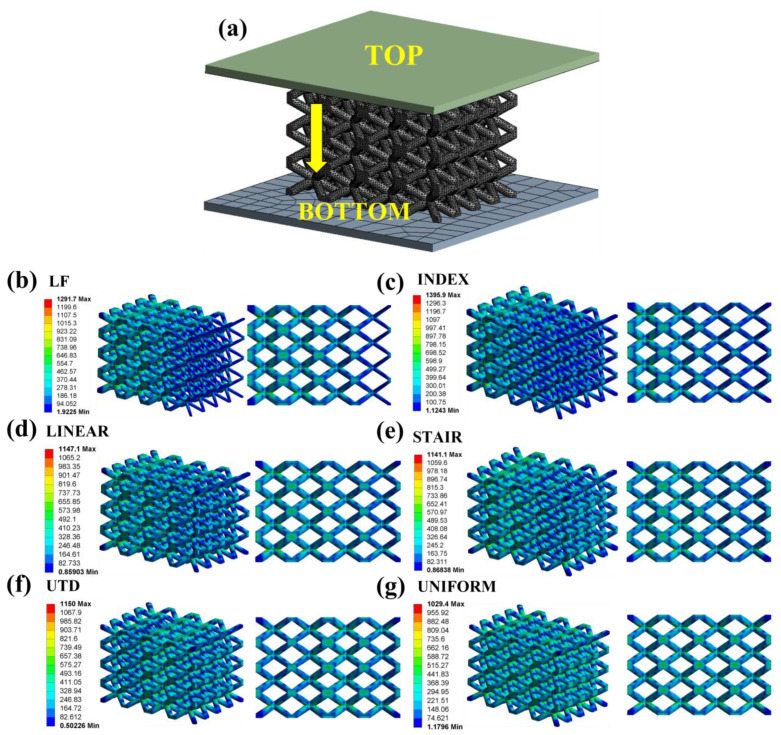
Pre-processing (**a**) and post-processing (**b**–**g**) of finite element analysis.

Finite element analysis of the lattice structure can more intuitively understand its stress distribution under a compression load. The numerical study was carried out on ANSYS Workbench software. A linear-elastic model was utilized for TC4 [48] (4430 Kg/m^3^ Density, 107 GPa Modulus, 1100 MPa Yield strength, 0.32 Poisson ratio). Both the top and bottom of the lattice structures are created as rigid body, and the friction coefficient between the two rigid bodies and the lattice structure is set to 0.2. As shown in Figure 9a, the rigid body at the top of the lattice structure is vertically displaced by 0.16 mm downward, except that all degrees of freedom are limited. All degrees of freedom of the rigid body at the bottom of the lattice structure are restricted. The models were meshed by four-node linear tetrahedral elements. After the mesh sensitivity test, each simulation contained between 776, 150, and 783,090 nodes. Elastic modulus ($E_{f}$) is calculated by finite element analysis based on Hooke’s law, as shown in Formula (5) [49].

(5) $$E_{f}=\frac{F_{r}}{A}/\frac{L_{0}}{L}$$

where $F_{r}$, A, $\;L_{0}$, L represent the reaction force of the bottom rigid body, the contact area between the top rigid body and the lattice structure, the displacement distance of the top rigid body, and the initial height of the lattice structure, respectively. It can be seen from the stress nephogram in Figure 9 that the stress modes of lattice structures with different density gradients are different, but the stress is mainly distributed at the joints of the struts. In addition, it can be seen from Figure 9b–d that the stress is mainly distributed in the parts with higher density. These results indicate that in the lattice structure with density gradient, the part with a higher density is the main bearing part. It can also be seen from the deformation behavior diagram of lattice structures under compressive loads (Figure 7) that the initial failure position of lattice structures with a logarithmic (LF), index (INDEX), and linear (LINEAR) density gradient occurs in the lower-left corner (the density of lattice structure decreases gradually from left to right in the direction shown in the figure). Similarly, it can be seen from the failure morphology diagram of the lattice structure after compression shown in Figure 8a that the location where the main failure occurs through the structure (near the connecting nodes of the struts) is consistent with the stress distribution location shown by the finite element analysis.

**Figure 10:**
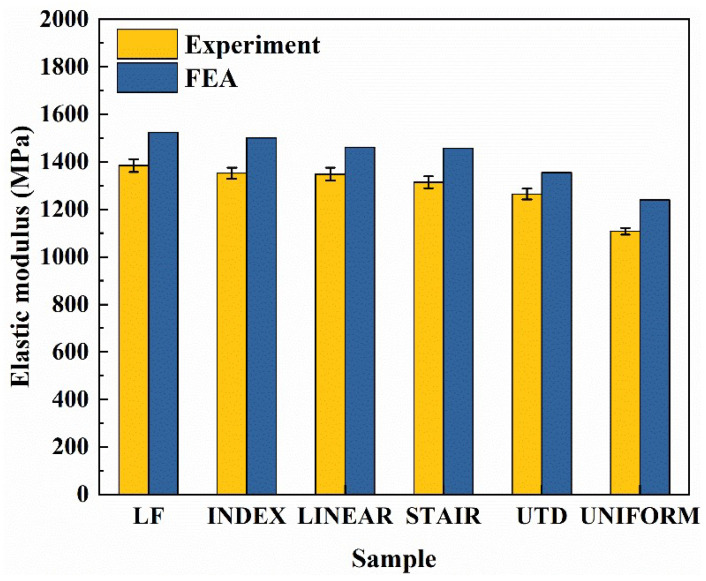
Comparison of elastic modulus between experimental data and finite element analysis.

The results in Figure 10 show that the elastic modulus of various lattice structures predicted by the finite element method is higher than the experimental value, which is predictable due to the forming characteristics of SLM. There will inevitably be some defects in the lattice structure in the SLM process [50,51,52,53,54], which will affect the mechanical properties of the lattice structure to a certain extent. However, in general, the maximum error is less than 11.9%, and the trend of elastic modulus of lattice structures with different density gradients predicted by the finite element method is equivalent to the experimental value. Therefore, in future lattice structure design, the finite element method can be used for preliminary prediction, which is of great significance for future lattice structure design.

## 4. Conclusions

In this study, in order to investigate the mechanical properties of BCC-based lattice structures with and without gradient in the direction perpendicular to the load under compressive loading, five lattice structures with different gradient variations with almost the same relative density were designed and fabricated using the SLM technique, and for comparison, uniform lattice structures with similar relative density were also fabricated. Using optical microscopy (OM) to observe the macroscopic morphology of the lattice structures and scanning electron microscopy (SEM) to observe the microscopic morphological features and fracture morphology of the lattice structures. In addition, the finite element analysis of different lattice structures was carried out. By analyzing the different lattice structures and the unit cell, the following conclusions were drawn:

1. The SEM and OM images clearly show that the BCC lattice structures with different density gradients can be fabricated by the adopted parameters. The surface morphology study shows that the large number of spherical particles attached to the lattice structure struts and the characteristics of the SLM fabrication process led to the increase in the strut diameter of the lattice structure, which results in the error between the actual relative density of the lattice structure and the designed relative density, but the maximum error does not exceed 2.88%;
2. Compression experiments show that the strength of lattice structures with gradients perpendicular to the loading direction is better than that of the uniform lattice structure. In particular, the elastic modulus of LF, the yield strength of LINEAR, and the first maximum compression strength of UTD are 29.25%, 17.45%, and 14.90% higher, respectively, than that of the UNIFORM. The energy absorption of the gradient lattice structure with exponentially increasing volume fraction (INDEX) is 45.74% higher than that of the uniform lattice structure;
3. The typical diagonal shear failure occurs for each lattice structure, and, compared to the uniform lattice structure, the INDEX, LINEAR, and STAIR lattice structures show crushing in both the top and bottom layers before diagonal shear failure occurs. Therefore, the INDEX, LINEAR, and STAIR lattice structures have better energy absorption capacity than the UNIFORM lattice structure. In general, the fracture morphologies of the lattice structures all exhibit dimples and smooth planes, indicating that the lattice structures exhibit a mixed brittle and ductile failure mechanism under compressive loading;
4. The results of finite element analysis show that in the lattice structure with a density gradient, the part with a higher density is the main bearing part. The larger the density difference between the two ends of the lattice structure, the larger the elastic modulus. In general, the stress is mainly concentrated at the joint of the struts, regardless of whether there is a difference in the density of the layer at both ends of the lattice structure. The inconsistency between the experimental results and the finite element prediction results can be attributed to the defects in the lattice structure caused by the forming characteristics of SLM.

## Figures and Tables

**Table 1:** Chemical composition of TC4 metal powder.

Element	Ti	Al	V	Fe	C	N	H	O
wt.%	Balance	5.5~6.5	3.5~4.5	0.25	0.08	0.03	0.0125	0.13

**Table 2:** The measurement of structural characterizations for the as-built lattice structures.

Sample	Relative Density (%)		Relative Error (%)
	Designed	Fabricated	
LF	26.195	26.777 ± 4.287	2.363 ± 1.639
INDEX	25.921	26.659 ± 3.512	2.876 ± 0.451
LINEAR	26.141	26.536 ± 4.381	1.512 ± 0.321
STAIR	26.134	26.635 ± 5.014	2.016 ± 0.173
UTD	26.095	26.518 ± 4.821	1.621 ± 0.851
UNIFORM	26.023	26.587 ± 3.906	2.170 ± 0.219

**Table 3:** Mechanical properties with different gradients of lattice structure as well as a uniform lattice structure.

Sample	First Maximum Compressive Strength (MPa)	Yield Strength (MPa)	Elastic Modulus (MPa)	Plateau Stress (MPa)	*p* Value
LF	81.01 ± 1.64	70.17 ± 2.25	1383.92 ± 27.14	63.61 ± 2.08	<0.001
INDEX	82.56 ± 1.59	68.70 ± 1.03	1352.57 ± 23.18	99.09 ± 2.13	
LINEAR	78.10 ± 1.52	71.51 ± 1.46	1348.27 ± 26.88	87.19 ± 2.66	
STAIR	78.32 ± 1.47	66.43 ± 2.41	1313.89 ± 25.05	55.14 ± 2.26	
UTD	81.76 ± 1.68	67.30 ± 1.57	1264.45 ± 23.16	72.20 ± 2.02	
UNIFORM	74.66 ± 1.14	63.78 ± 1.29	1107.79 ± 13.89	71.49 ± 1.10	

**Table 4:** Densification onset strain and energy absorption properties of different lattice structures.

Sample	Densification Onset Strain	Energy Absorption per Unit Volume (MJ/m^3^)	Energy Absorption Efficiency (%)
LF	0.408 ± 0.036	23.79 ± 1.34	29.57 ± 1.46
INDEX	0.387 ± 0.046	33.48 ± 1.28	33.15 ± 1.48
LINEAR	0.426 ± 0.028	34.62 ± 2.63	30.42 ± 2.16
STAIR	0.451 ± 0.021	25.97 ± 2.64	36.17 ± 1.48
UTD	0.443 ± 0.036	28.16 ± 1.80	30.97 ± 1.89
UNIFORM	0.403 ± 0.012	26.28 ± 1.16	29.24 ± 1.54

## Data Availability

The raw/processed data required to reproduce these findings cannot be shared at this time as the data also form part of an ongoing study.
